# Perioperative Observations and Outcome in Surgical Treatment of Malignant Peripheral Nerve Sheath Tumors

**DOI:** 10.3390/cancers16223757

**Published:** 2024-11-07

**Authors:** Julian Zipfel, Jonas Tellermann, Kevin Paul Ferraris, Florian Grimm, Antje Bornemann, Benjamin Bender, Helmut Dittmann, Jürgen Schäfer, Konstantin Nikolaou, Ruth Ladurner, Volker Steger, Marcos Tatagiba, Martin U. Schuhmann, Isabel Gugel

**Affiliations:** 1 Department of Neurosurgery, University Hospital Tübingen, BW 72076 Tübingen, Germany; 2 Centre of Neurofibromatosis and Rare Diseases, University Hospital Tübingen, BW 72076 Tübingen, Germany; 3 Section of Pediatric Neurosurgery, University Hospital Tübingen, BW 72076 Tübingen, Germany; 4 Division of Pediatric Neurosurgery, BC Children’s Hospital, University of British Columbia, Vancouver, BC V6H 3N1, Canada; 5 Department of Pathology and Neuropathology and Comprehensive Cancer Center, University Hospital Tübingen, BW 72076 Tübingen, Germany; 6 Department of Diagnostic and Interventional Neuroradiology, University Hospital Tübingen, BW 72076 Tübingen, Germany; 7 Department of Nuclear Medicine and Clinical Molecular Imaging, University Hospital Tübingen, BW 72076 Tübingen, Germany; 8 Department of Diagnostic and Interventional Radiology, University Hospital Tübingen, BW 72076 Tübingen, Germany; 9 Department of General, Visceral and Transplantation Surgery, University Hospital Tübingen, BW 72076 Tübingen, Germany; 10 Institute of Clinical Anatomy and Cell Analysis, University of Tübingen, BW 72076 Tübingen, Germany; 11 Department of Thoracic and Cardiovascular Surgery, German Cardiac Competence Center, University Hospital Tübingen, BW 72076 Tübingen, Germany

**Keywords:** neurofibroma, neurofibromatosis type 1, surgery, malignant peripheral nerve sheath tumor, MIB-1, FDG-PET

## Abstract

Background/Objectives: This retrospective observational study aimed to investigate the perioperative outcome in Malignant Peripheral Nerve Sheath Tumors (MPNSTs) with and without relation to Neurofibromatosis Type 1 (NF1) and to detect possible influencing factors. Methods: Clinical reports, histopathological evaluations, imaging, and treatment characteristics were reviewed in 35 operated MPNSTs in 33 patients. Possible predictive valuables included disease type, preoperative tumor volume, SUV and MIB-1 proliferation index, resection margins, the presence of metastasis, and whether radio-/chemotherapy was received. Results: Patients with NF1 were younger (mean age: 29 ± 13, 8–54 years) than sporadic cases (mean age: 45 ± 13, 24–67 years) and exhibited significantly larger preoperative tumor volumes (mean 299 vs. 18 cm^3^, *p* = 0.048). Most tumors were located in the facial/cervical/neck area (34%, n = 12), followed by the trunk (31%, n = 11), lower extremity (17%, n = 6), upper extremity (14%, n = 5), and intraspinal area (3%, n = 1). NF1-associated MPNSTs appeared predominantly on the trunk (39%) and sporadically in the facial/cervical/neck area (50%). Complete resection was possible in 66% and an improvement in or stability of function was achieved in most cases (motor 69%, sensory 74%), as well as a decrease in pain intensity (63%). NF1-associated MPNSTs exhibited more severe pain scores (median VRS scale 2, *p* = 0.002) compared to sporadic tumors (median VRS scale 0.5). Sporadic MPNSTs located at the head/facial/brachial plexus and upper extremities exhibited better preoperative functions compared to those on the lower extremities. In 12 cases with available [^18^F]FDG PET, the mean preoperative SUV (9.8 ± 7.2) positively correlated with the mean maximum MIB-1 index (34 ± 26%, *p* = 0.005) and the mean preoperative tumor volume (474.7 ± 68.6 cm^3^, *p* = 0.047). The overall survival (OS) was significantly longer in tumors with higher resection extents (R0, *p* = 0.01) and without accompanying metastasis (*p* = 0.046), and tended to be longer, but not significantly so, in sporadic MPNSTs. In six and seven tumors, with R1/R2 resection margins and present metastasis, respectively, solid or combined neo-/adjuvant radio-/chemotherapy led to a significantly shorter OS (*p* = 0.014). Conclusions: NF1-associated MPNSTs have larger tumor volumes, higher SUVs and MIB-1 proliferation indices, and a shorter overall survival period. Nevertheless, surgery can improve symptoms, particularly medication-resistant pain, and should also be considered in advanced disease for symptom control/improvement.

## Simple Summary

Malignant peripheral nerve sheath tumors (MPNSTs) are a diagnostic and therapeutic challenge requiring interdisciplinary teamwork. With this study, we aimed to investigate the perioperative outcomes in patients with these tumors. We demonstrated that an increase in the levels of SUVs on preoperative [^18^F]FDG PET and large tumor volumes may be a predictive marker for highly proliferative tumors with a poor prognosis, particularly in patients with underlying Neurofibromatosis type 1. Surgery for MPNSTs can improve symptoms, particularly medication-resistant pain, and should also be considered in advanced disease for symptom control/improvement.

## 1. Introduction

Malignant peripheral nerve sheath tumors (MPNSTs) are malignant, locally aggressive soft-tissue sarcomas with a predisposition to metastasize. They rarely occur in the general population (incidence 1:100,000) but patients with neurofibromatosis type 1 (NF1) are at an approximately 10% risk (incidence 1:3500) of developing such malignancies over their lifetime [1]. Among all MPNST cases, 50% are associated with NF1 disease [1,2], 10% exhibit a previous radiation exposure [3], and the remaining are sporadic [4]. A clinical distinction between benign and malignant peripheral nerve sheath lesions is often not possible due to the unspecific and similar symptoms (pain, neurological impairment, and motor dysfunction, as well as disfigurement) [3,5]. Since plexiform neurofibromas (PN) are usually detected during childhood and show the greatest growth dynamics, the growth and progression of PN in adulthood should always raise suspicion of malignancy [6].

The treatment of these tumors is often multidisciplinary and includes surgical resection and adjuvant radiation for localized high-grade MPNSTs, whereas chemotherapy is administered in those with metastatic MPNSTs. Nevertheless, the prognosis remains poor; the 5-year local recurrence rates are high (27–86%) [5,7,8] and the 5-year overall survival rates are low (21–52%) [8,9,10]. Prognostically unfavorable factors are large tumor volumes [3,5,8], metastasis at initial diagnosis [8,11,12], recurrence [3], and the relation to NF1 disease [8] and histopathological grading [13].

In patients with NF1, MPNSTs characteristically begin as plexiform neurofibroma (PN) and atypical neurofibromatous neoplasm of unknown biological potential (ANNUBP), which are considered precancerous lesions [14]. ANNUBP is an NF1-associated tumor with the histologic features of nuclear atypia, hypercellularity, and increased mitotic activity [15]. Although its malignant potential remains uncertain, the recurrence rates after radical surgery are low and there is no risk of metastasis [15]. Since ANNUBP typically shows an increase in FDG uptake on PET-CT/MRI, early detection and surgical treatment confer a chance of preventing a further transformation to MPNSTs [15].

In this retrospective analysis, we investigated the perioperative outcome, as well as the influence and value of the FDG PET uptake (SUV) and MIB-1 proliferation index, in the context of NF1-associated and sporadic MPNSTs.

## 2. Materials and Methods

### 2.1. Patients and Clinical Data

A total of 33 patients with either NF1 (21 patients) or sporadic (12 patients) and 35 histologically confirmed MPNSTs (NF1 n = 25; sporadic n = 12) were included in this retrospective analysis. Among them, two (of 25) NF1-associated tumors were recurrent tumors. All patients who were operated on and followed up between 2005 and 2023 at the Department of Neurosurgery and Centre of Neurofibromatosis and Rare Diseases, University Hospital Tübingen, were included. The diagnosis of NF1 was established using the published consensus criteria [16]. None of the patients exhibited previous radiation exposure in the investigated tumor lesions. Data from six tumors from six pediatric patients (four patients with NF1 and two sporadic cases) were part of a prior (published) study and were included in this cohort (Zipfel et al. [17]).

In 10 NF1 patients, a molecular genetic analysis was carried out due to a lack of typical diagnostic criteria, the patient or family having the desire to have children, or clinical signs of the presence of an NF1 microdeletion syndrome. One sporadic patient was also molecularly tested to exclude NF1 disease.

Patients’ perioperative clinical evaluation included a full medical history, general physical, and symptom-based neurologic examination directly before and after surgery as part of the inpatient stay and at the final clinical follow-up in our NF Center. In addition, the cumulative clinical status (equal, improved, worsened) regarding any change to one of the three main categories at the last follow-up examination and the overall survival was evaluated. For the classification of the clinical function, we used the Medical Research Council Scale for Muscle Strength (MCR) [18], the Sensory Rating Scale (SRS) [19], and a four-point Verbal Rating Scale (VRS, 0–3) with the words “no pain” (0), “slight pain” (1), “moderate pain” (2), and “severe pain” (3) to measure pain intensity [20]. Pain impression was only evaluated for the surgical outcome, not for the clinical or differential characterization, and was attributable to the palpable, imaging, or sonographic neurofibroma/MPNST findings. It occurred either acutely or was chronically progressive over time and was dermatome-related in the form of radiating pain.

A radiological evaluation was performed, either by MRI of the target region/whole-body MRI and/or in the form of a peripheral nerve ultrasound examination. In the case of well-defined lesions that allowed for good accessibility for ultrasound testing, not all patients underwent preoperative MRI. An 18F-fluorodeoxyglucose (FDG) PET/CT or MRI was completed in 12 (10 NF1 and 2 sporadic) patients in whom malignancy was suspected based on conventional MRI criteria (rapid tumor growth, large lesions, inhomogeneous signal intensity “target” sign in the conventional MRI).

**Figure 1:**
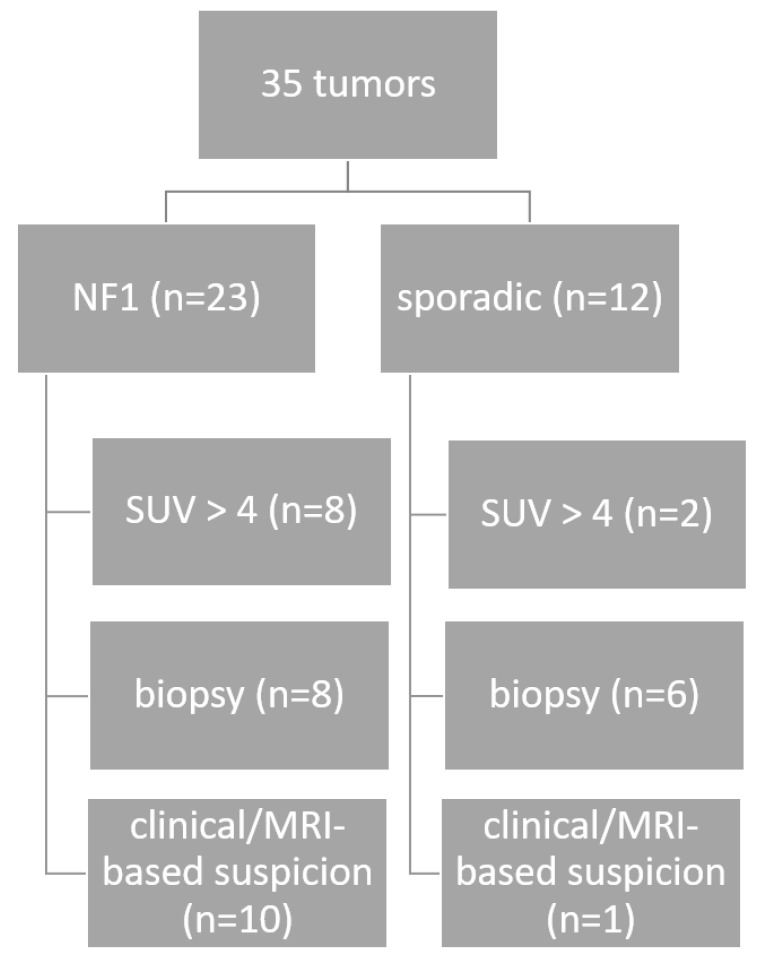
Diagnostic work-up until surgery for the 35 MPNSTs.

In patients with available MRI, and for the determination of the preoperative tumor volume, T2-STIR and thin-sliced (<3 mm) sequences in axial layering were uploaded into the iPlan Net software (v 2.0, Brainlab, Feldkirchen, Germany) and volumes were measured using the manual segmentation tool. Tumors that did not meet these criteria or had inadequate MRI quality (e.g., due to severe artifacts caused by patient motion) were excluded from volumetry. The preoperative MRI was no older than 4 weeks. The diagnostic work-up is illustrated in Figure 1.

The indications for surgery or primary open, sonographic- or radiological-guided (needle) biopsy were as follows:

(1) Persisting and drug-resistant pain;
(2) Tumor growth progression in known tumors (clinically in visible or palpable lesions or radiologically by ultrasound or MRI for deeper lesions);
(3) Development or worsening of known focal neurological deficits;
(4) Clinical and/or radiological suspicion of a malignant transformation as described beforehand, if a PET MRI/CT was available/performed;
(5) Increased SUVs > 4 in FDG PET in known symptomatic or asymptomatic tumor lesions.

Resection was subsequently performed in eight NF1-associated and six sporadic MPNSTs with preoperative biopsy. Microsurgical removal was performed following sarcoma criteria and according to the R0/R1 Classification System (R0 = no residual tumor; R1 = microscopic residual tumor; R2 = macroscopic residual tumor) [21]. Partial tumor resection (R2) was only carried out in cases of confirmed poor oncological prognosis (e.g., the presence of metastases) or non-operable conditions (e.g., invasion of vital structures/vessels). Intraoperative neurophysiological monitoring and/or direct nerve stimulation were used to identify functional nerve fibers to minimize the risk of functional loss and to guide the extent of resectioning, particularly in large, diffuse tumors with a poor prognosis and the surgical intent of pain relief or functional stability/improvement.

An intraoperative pathologist consultation (IC) was performed to guide immediate surgical management.

All tumors were histologically confirmed as MPNSTs and classified as either low-grade or high-grade MPNSTs [15]. As a proliferation marker, the MIB-1 index was also determined as a percentage as part of the routine histological diagnostics and taken into account in the following analysis.

### 2.2. Data Evaluation

The statistical analysis was performed using SPSS (IBM SPSS Statistic for Windows, Version 22.0., IBM Corp., Armonk, NY, USA).

A linear regression was run to assess the relationship between the histopathological proliferation rate (MIB-1 index in %) and preoperative tumor volume. In 12 mixed cases (2 sporadic and 10 NF1-associated MPNSTs) with available preoperative FDG PET, a linear regression model was used to assess the relationship between the histopathological proliferation rate and preoperative volume, as well as SUVs.

A Mann–Whitney U test was run for each NF1- and SPO-associated MPNST to determine if there were differences in the independent variables (motor and sensory function and pain) between the grading of tumors (“high-grade” vs. “low-grade”) (“NF1” vs. “sporadic”).

A Kruskal–Wallis H test was run to determine if there were differences in motor, sensory, and pain rating scales between the five location categories (see Table 1) in NF1- and SPO-related MPNSTs. The distributions of the rating scales were not similar for all groups, as assessed through the visual inspection of a boxplot.

A Mann–Whitney U and Wilcoxon signed-ranked test were run to determine if there were differences in the independent variables (pre- and postoperative MCR, SRS, VRS, and preoperative volume and proliferation rate) between tumors related to NF1 (“NF1 associated”) and sporadic cases (“SPO”), as well as between different tumor gradings (“high-grade” vs. “low-grade” MPNSTs). 

A Kaplan–Meier curve was used to visualize the overall survival (OS) between the comparisons of NF1 vs. sporadic, resection margins (R0–R2), the presence of metastasis, and patients with/without solid or combined (neo-) adjuvant radio-/chemotherapy. Here, OS was defined as the period between the date at the time of surgery until the date of death (in months). This included OS rates in the different comparison groups and the corresponding 95% CI, as well as the mean OS and corresponding CI. Patients who did not die during the observation period were censored at the date of the last clinical follow-up evaluation.

## 3. Results

### 3.1. Patients, Tumors, and Clinics

Detailed demographic and clinical data are summarized in Table 1 and Supplementary Tables S1–S3.

Of all operated MPNSTs, the majority of cases were located in the facial/cervical area (34%, n = 12), closely followed by the trunk location (31%, n = 11), and further, in descending order, in the lower extremity (17%, n = 6), upper extremity (14%, n = 5), and lastly the intraspinal area (3%, n = 1). NF1-associated MPNSTs clearly predominantly appeared on the trunk (39%) and sporadic MPNSTs appeared in the facial/cervical area (50%).

No mosaic cases were identified and in three NF1s and one sporadic patient, no mutation could be detected in the blood or in tumor DNA.

In all NF1 patients, the lesions were primarily determined either clinically, through a visual inspection, or via radiological monitoring (ultrasound or MRI); however, at the beginning of the monitoring they appeared to be uncritical, of a small size, and without neurological symptoms.

The most common indication for surgery for NF1 was the suspicion of malignancy in 39% (n = 9) and sporadic MPNST growth progression in 33% (n = 4). This was followed by pain in 26% (n = 6) and tumor growth progression in 22% (n = 5) in NF1-associated MPNSTs versus pain, as well as focal neurological deficits in 25% each (n = 3) in sporadic MPNSTs. Focal neurological deficits were less common in NF1 (13%, n = 3). Malignancy was clinically suspected in one sporadic case. FDG PET identified an additional two NF1s and one sporadic case with elevated SUV, indicating an increased glucose metabolism and thus suspicion of malignancy.

However, taking into account the low numbers obtained by the two PET scans in sporadic cases, these showed a lower SUV than the NF1-associated tumors. Preoperative tumor volumes were significantly (*p* = 0.048) higher in NF1-associated tumors (mean volume 299.1 cm^3^) compared with sporadic cases (mean volume 17.8 cm^3^). Except for one NF1 patient with a late presentation at our department and a progressive disease (Supplementary Table S2, case 16, metastasis, SUV 25.6) and a partially performed resection (R2), all tumors with preoperatively increased SUVs (>4) in FDG PET were completely resected (R0).

The majority, 60% (n = 21), of all tumors (NF1 and SPO) were histologically classified as high-grade MPNSTs.

In most cases (66%, n = 23), total resection with healthy surrounding tissue or with nerve (R0) was achieved, whereas five tumors (14%) were totally macroscopically resected, and the microscopic tumor contamination of margins was observed (R1). Only seven cases (20%) with a known poor prognosis or non-operable lesions had a macroscopic tumor residual. R2 resection occurred in these cases for the following reasons (please refer to Supplementary Table S2):

(1) Progressive and palliative situation due to multiple metastases; thus, surgical intervention was required to stop/prevent focal neurological deficits (cases 2, 8, 16).
(2) Initial partial resection under functional preservation criteria (cases 25, 27) with secondary total removal after histological MPNST confirmation.
(3) Inoperability due to the local/surrounding infiltration of critical structures (e.g., vessels, bone; cases 8, 19).

Among the seven NF1-associated and five sporadic cases with a resection grade R1/2, three patients were deceased by the end of the observation period for each diseased type.

Metastasis (cerebral, hepatic, osseous, pulmonary, meningeal, lymph nodes) occurred in seven NF1-associated (30%) and three sporadic (25%) MPNST cases. Among them, five NF1-associated and two sporadic cases died by the end of the observation period due to MPNST-related disease.

Neo-/adjuvant radiation treatment was carried out in seven (30%) NF1-associated and two (17%) sporadic, and neo-/adjuvant systemic treatment was carried out in eight (35%) NF1-associated and five (42%) sporadic cases.

Directly after surgery, motor function was improved in 26% (n = 6)/0%, maintained in 48% (n = 11)/58% (n = 7), and worsened in 26% (n = 6)/42% (n = 5) patients with NF1-associated/SPO tumors (Supplementary Table S1). Postoperative sensory function improved in 22% (n = 5)/0%, remained stable in 52% (n = 12)/75% (n = 9), and decreased in 26% (n = 6)/25% (n = 3) of NF1-associated/SPO cases.

Lastly, pain intensity improved in 78% (n = 18)/33% (n = 4), was maintained in 22% (n = 5)/67% (n = 8), and worsened in 0% of NF1-associated/SPO tumor cases.

Of a total of 31 (35) tumors, the last documented clinical follow-up after surgery in our NF center (mean 38 ± 43, range 0–165 months) showed a stable clinical status in 16 tumors (52%), an improvement in 4 tumors (13%) and a deterioration in 11 tumors (35%) compared to the clinical status directly after surgery. For the remaining four tumors, no further information on follow-up after discharge was available.

Overall, the complication rate was very low; 34 cases had an uneventful peri- and postoperative course (97%), and only one NF1 case (3%) developed a postoperative cerebrospinal fluid fistula, which was successfully treated by lumbar drain. Ten NF1 and four sporadic patients died due to complications directly related to their MPNST disease by the end of the observation period.

### 3.2. Correlation Between Maximum Histopathological MIB-1 (Proliferation) Index (%), FDG PET SUV, and Preoperative Volume for All Tumors

A linear regression was run to understand the relationship between preoperative tumor volume and MIB1-proliferation rate in all tumors. For this, the maximum proliferation rate was considered. To assess linearity, a scatterplot was plotted (Supplementary Figure S1). Visual inspection indicated a linear relationship between the variables. There was homoscedasticity and normality among the residuals. Three outliers (preoperative volume 2294.3 cm^3^, 908.3 cm^3^, and 844 cm^3^) were identified. The analysis was run with and without them. Preoperative tumor volume (mean 211.2 ± 434.7, 1.2–2294.3 cm^3^) statistically significantly predicted the maximum MIB-1 proliferation rate (mean 30 ± 23, 3–80%); F (1.26) = 4.888; *p* = 0.036.

For the 12 cases with available FDG PET (10 NF1 and 2 sporadic cases), a Pearson correlation test was run to assess the relationship between the MIB-1 proliferation rate and SUV, as well as preoperative tumor volume. Because of the low number, no distinctions between NF1 and sporadic cases and between MRI and CT were made.

Residuals were independent, as assessed by a Durbin–Watson Statistic of 1.518. The maximum MIB-1 proliferation rate (34 ± 26%) was significantly positively correlated with the SUV (mean 9.8 ± 7.2, *p* = 0.005) and with the preoperative tumor volume (mean 474.7 ± 68.6 cm^3^, *p* = 0.047).

### 3.3. Association Between Histopathological Grading (High- vs. Low-Grade), Disease Type (NF1-Associated vs. Sporadic), and Parameters

Distributions of the motor (MRC), sensory (SRS), and pain (VRS) rating scores, as well as preoperative volume and proliferation rate, were similar in the groups “high-grade” and “low-grade” and in the groups “NF1-associated” and “sporadic”, as assessed by visual inspection.

The mean values for the maximum proliferation rate (in %) for “high-grade” (39 ± 24.62, range 3–80%) were significantly higher (*p* = 0.020) compared with “low-grade” MPNSTs (18 ± 15.48, range 3–50%).

The median values for preoperative VRS, as well as the mean values for preoperative volume, differed significantly (*p* = 0.002 and *p* < 0.001) between the groups “NF1” vs. “sporadic” MPNSTs. Therefore, NF1 cases exhibited a higher preoperative pain intensity score and higher preoperative tumor volumes when compared with sporadic cases.

All other comparison parameters did not reach statistical significance in the comparison groups. Detailed values are outlined in Table 2.

### 3.4. Location Category Distribution of NF1 and SPO-Related MPNSTs and Association with Parameters

There was no significant difference in all observed location categories among NF1-associated MPNST cases (*p* > 0.05).

**Figure 2:**
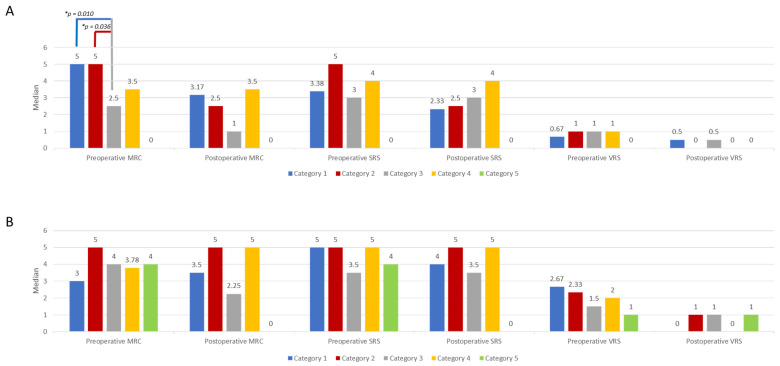
Group differences in pre- and postoperative motor and sensory function and pain intensity in (**A**) sporadic (significant) and (**B**) NF1-associated (non-significant) MPNSTs; * = *p* < 0.05.

Among sporadic MPNSTs, the median values for “preoperative MCR” (H(3) = 8.033, *p* = 0.045) differed significantly between the categories. Subsequently, pairwise comparisons were performed using Dunn’s (1964) procedure with a Bonferroni correction for multiple comparisons. This post hoc analysis revealed no statistically significant group differences (adjusted *p*-value > 0.05). For the sporadic and NF1-associated MPNST cases, group differences are illustrated and highlighted in Figure 2 (* adjusted *p*-values).

No significant group differences were observed in NF1-associated MPNSTs in any of the functional categories/pain categories. The categories were as follows. 1: head/face/neck/brachial plexus; 2: upper extremity; 3: lower extremity; 4: trunk (thorax/abdomen/pelvic/back); 5: intraspinal. There was no intraspinal MPNST location among the sporadic MPNSTs.

The sporadic cases in the functional categories for preoperative MRC (categories 1 and 2; median = 5), preoperative VRS (categories 2, 3, and 4; median = 1), and postoperative VRS (categories 1 and 3; median = 0.5; categories 2, and 4; median = 0) exhibited equal values and no differences.

This was also the case for the NF1-associated cases with the same values in the different categories. There were no differences for these cases either. In NF1-associated tumors, good pain relief/improvement could be achieved through surgery in lesions located at the neck/brachial plexus, at the torso, and at the upper extremity. NF1-associated tumors of the lower extremity exhibited worse neurological outcomes compared with lesions of the upper extremity, with a particular worsening of motor function after surgery.

Overall, sporadic cases tended to have a worse postoperative neurological outcome and less perioperative pain compared with NF1-associated cases. MPNSTs of both disease types located at the torso seemed to be relatively stable, with postoperative improvements in neurological function and improvements in pain intensity/relief.

### 3.5. Overall Survival Between Comparisons

**Figure 3:**
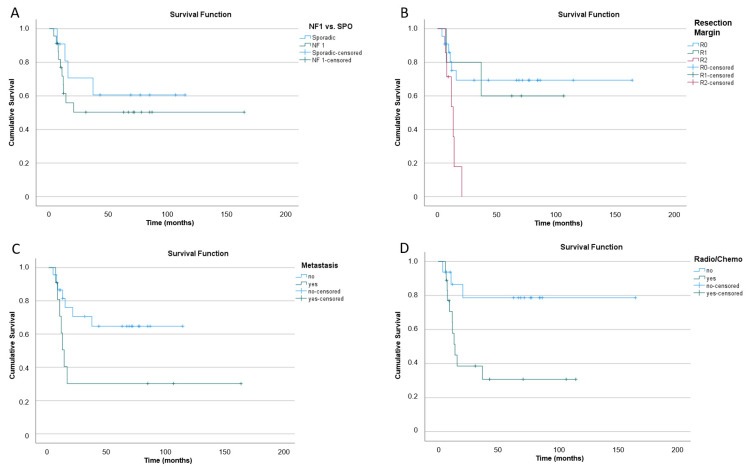
Overall (cumulative) survival in the following comparisons: (**A**) NF1 vs. sporadic (SPO), (**B**) different resection extents (R0–R2), (**C**) with or without metastasis, and (**D**) solid or combined neoadjuvant or adjuvant radio-/chemotherapy.

A long rank test was run to determine if there were differences in the survival distribution for the different comparisons. The survival distributions were statistically significantly different regarding the resection extent (R0-R2; X^2^(2) = 9.143, *p* = 0.010, Figure 3B), presence of metastasis (X^2^(1) = 3.983, *p* = 0.046, Figure 3C), and (neo-) adjuvant radio-/chemotherapy (X^2^(1) = 6.085, *p* = 0.014, Figure 3D). No statistical significance was achieved regarding the survival distribution between NF1 vs. sporadic, but a tendency towards a shortened overall survival (OS) was seen for NF1 patients compared to patients with sporadic tumors (Figure 3A). A longer mean overall survival was therefore seen in tumors with higher resection margins (R0: 95% CI, mean OS = 117 ± 16 range 85–149 months; R1: 95% CI, mean OS = 73 ± 19 range 36–110 months; R2: 95% CI mean OS = 13 ± 2 range 9–17 months), patients without metastasis (95% CI, mean OS = 79 ± 11 range 59–101 months versus (with metastasis) mean OS = 57 ± 22 range 13–101 months), and patients that did not undergo a solid or combined neo-/adjuvant radio-/chemotherapy (95% CI, mean OS = 132 ± 17 range 100–165 months versus (with radio-/chemotherapy) mean OS = 45 ± 12 range 21–69 months). Patients with sporadic MPNSTs had a longer mean overall survival of 77 ± 15 months (95% CI, 48 to 106 months) compared to NF1 cases (95% CI, mean OS = 88 ± 17, range 54–123 months.

## 4. Discussion

In this study, we investigated the perioperative clinical outcome of MPNSTs with and without NF1 disease. We also examined possible influencing factors for postoperative outcomes, such as tumor location and volume, histopathological grading, SUV, and MIB-1 proliferation index.

Like other studies, we were able to show that MPNSTs in NF1 patients appear earlier, in the second to fourth decades of life (in our cohort, mean age in NF1 was 29 vs. 45 years in sporadic MPNST) [23], have a larger preoperative volume (in our cohort, mean volume in NF1 was 299 cm^3^ vs. 18 cm^3^ in sporadic MPNST) [3], and are similar to our NF1-related cases, which were predominantly located at the trunk [8]. On the other hand, sporadic cases were more frequently (50%) located at the head/neck, facial, and brachial plexus areas.

A slight predominance (60%) of NF1-related MPNSTs was seen in our cohort, but this was because our center is an NF reference center.

Although the resection extent was not too high overall (66% R0), we could achieve an improvement in or stability of sensory and motor function in the majority of cases for both disease types (motor 69%, sensory 74%), and improvement in/stability of pain sensation (100%).

Functional outcomes appear to depend strongly on tumor location. Lesions at the trunk, which are mostly of non-functional relevance, can be operated on more radically than lesions at the brachial plexus, for example. Thus, a better functional outcome can be obtained via surgical resection of the former. In contrast, surgical resection of the latter, which necessitates complete resection in the framework of the sarcoma criteria for MPNSTs, often entails a functional loss due to the necessity of neurectomy.

As patients with MPNSTs often suffer from severe pain, the postoperative improvement rate of 63% is encouraging. For those cases with a known poor prognosis (e.g., inoperable (widespread) metastasis [8,11,12], recurrent tumors [3], histopathological (high-) grading [13], or a large tumor size [3,5,8]), function-preserving partial resection could be an option for pain relief. In our cohort, patients with NF1 exhibited severe pain scores compared to sporadic ones. The location had no significant influence on perioperative outcomes in NF1-related MPNSTs. Sporadic cases exhibited a better preoperative function in lesions located at the head/facial/brachial plexus and upper extremity compared to the lower extremity. Other studies could confirm a significant difference among the two groups in terms of tumor lesion sites [8].

FDG PET CT/MRI is invaluable in detecting the onset of malignancy. This is of special value for NF1 cohorts who might have a high inner tumor load or complex and invasive plexiform neurofibroma lesions, and are therefore at a high risk of malignant transformation to MPNSTs at about 10% over their lifetime [1,24]. However, the routine use of PET CT/MRI is not commonplace. Reinert et al. [10] demonstrated the clinical utility of PET imaging in detecting malignant transformations among patients with NFI. They found that SUV thresholds in MPNSTs were significantly higher compared to those in neurofibromas [10]. An SUVmax of ≥3.5 is widely used in the literature as a cut-off point for malignant transformation into MPNSTs [10,25,26]. Our data show that the SUV correlates with the proliferative index marker MIB-1 (Ki-67) and a large tumor volume—two factors that are prognostic for the presence of malignancy in other studies [27]. It appears that the higher the SUV in FDG PET, the higher the MIB-1 index in the surgically resected MPNSTs. An MIB-1-index >10% may indicate an MPNST, [27] while eight tumors in our cohort (22.9%) showed lower values.

In our cohort, PET imaging was mainly performed in patients with NF1 in whom malignancy was suspected based on conventional MRI criteria (12 patients). In the other 10 NF1-associated cases, there were no typical conventional MRI criteria suggestive of malignancy (rapid tumor growth, large lesions, or the inhomogeneous signal intensity “target sign” in the MRI). This demonstrates the importance and suitability of using PET imaging in the routine follow-up of patients with NF1 in whom a higher risk of developing MPNSTs might be expected, such as those with the whole-gene deletions of NF1 [28], a high tumor burden including a large number of/or expansive plexiform neurofibroma, and large or rapidly growing tumor lesions [29]. For these clinical scenarios, it may be valuable to integrate PET diagnostics into the routine follow-up early on rather than during a later time interval once the conventional MRI criteria for malignization have been met. Depending on the initial metabolic activity, this should then be repeated during the disease follow-up. At this point, lesions with an SUV in FDG PET of 3–4 [10,26] should be removed or biopsied. The surgical therapy of MPNSTs should ideally achieve complete removal with negative tumor margins. Adjuvant radiotherapy may improve local control [3].

Interestingly, in our cohort, patients who received solid or combined neo-/adjuvant radio-/chemotherapy showed a lower overall survival compared to patients who did not undergo this therapy. This can probably be explained by the fact that, among them, in six and seven tumors, only an R1/R2 resection was performed, respectively, and metastasis was already present; therefore, further therapy was necessary. Consequently, and similar to other studies, patients with metastasis and positive surgical margins showed a significantly lower overall survival period [7]. The same applies to NF1 patients with MPNSTs, who had lower survival rates [7], which we could also see in our cohort, although our results were not significant.

The limitations of this study arise from the retrospective character of the analysis. Furthermore, NF2 and SWNT are rare genetic conditions, and while the presented data set is the largest to date, the statistical power of our study is limited. No dynamic parameters of FDG uptake were evaluated, only static SUVs.

At the time of MPNST diagnosis, 15 patients with NF1 (71%) presented for the first time at our NF center, who were not previously affiliated with any other NF/pediatric center. Only two patients were undergoing regular controls in an NF center. At that time, their disease had already progressed, with corresponding severe symptoms and a large tumor volume, which is reflected in the elevated rate of high-grade tumors.

In the remaining four patients, compliance was poor, resulting in long control breaks (5–6 years) due to cognitive or behavioral comorbidities. This represents a relevant gap in care, especially for adults who outgrow the very detailed and reliable pediatric checks, and especially given the lack of knowledge among primary care providers, who are rarely confronted with this clinical picture. As PET is not generally reimbursed in our country, there may also be delays in cost approval arrangements for PET imaging in some cases (~2–3 years), which in turn precludes the timely treatment planning that is indispensable for extensive yet primarily non-operable tumors. Early presentation to an NF/pediatric referral center, as well as social support, are therefore crucially needed in these cases. Furthermore, patient education on the identification of suspicious lesions is important so that the development of new neurological symptoms, pain, and/or the enlargement of an existing plexiform neurofibroma will always raise suspicion of malignant transformation and facilitate early treatment-seeking behaviors [16].

## 5. Conclusions

The management of both NF1-associated and sporadic MPNSTs poses a diagnostic and therapeutic challenge, leading to high morbidity and mortality, and poor outcomes. As there is no effective targeted therapy at present, surgical removal remains the mainstay treatment. Surgery can also improve symptoms and is particularly important for medication-resistant pain, which is a common symptom in MPNSTs. The early detection of these lesions is crucial for favorable outcomes and survival. FDG PET is an indispensable tool for detecting lesions at an early stage and for operative treatment planning, in addition to helping to determine whether there is a high proliferation index—a prognostic marker for aggressiveness and survival. Therefore, the integration of PET early on during the clinical follow-up should be considered, particularly for patients with a known high tumor load and a large lesional size in the context of NF1.

## Figures and Tables

**Table 1:** Demographic data of 35 operated malignant peripheral nerve sheath tumors in 33 patients.

	NF1	SPO
No. of patients/tumors	21/23	12/12
Sex (no. of females/males)	7/14	5/7
Total follow-up in months (mean ± SD, range)	34 ± 42, 1–165	43 ± 39, 1–114
Age at time of surgery in years (mean ± SD, range)	29 ± 13, 8–54	45 ± 13, 24–67
Family history of NF1 (yes/no)	7/14	0/0
Detected NF1-related gene mutation types	No of patients	
- Missense mutations;	2	-
- Nonsense mutations;	2	-
- Splicing mutations;	2	-
- Deletion.	1	-
No mutation detected	3	1
Not examined	11	11
Location Categories of peripheral (1–4) and intraspinal (5) MPNSTs	%, n = number of tumors	
- 1: Head/face/neck/brachial plexus;	26%, n = 6	50%, n = 6
- 2: Upper extremity;	13%, n = 3	17%, n = 2
- 3: Lower extremity;	17%, n = 4	17%, n = 2
- 4: Trunk (thorax/abdomen/pelvic/back);	39%, n = 9	17%, n = 2
- 5: intraspinal.	4%, n = 1	0
Indication for surgery	%, n = number of tumors	
Persisting/drug-resistant/progressive pain;	26%, n = 6	25%, n = 3
- Tumor growth progression;	22%, n = 5	33%, n = 4
- Focal neurological deficits;	13%, n = 3	25%, n = 3
- Suspicion of malignancy *;	39%, n = 9	17%, n = 2
- Increased SUV **.	9%, n = 2	8%, n = 1
Preoperative tumor volume in cm^3^ (mean ± SD, range) *p*-value	299.1 ± 500, 11.5–2294.3 0.048	17.8 ± 15.46, 1.2–44.9
Preoperative SUV in FDG PET (mean ± SD, range) No of PET MRI/CT scans	9.8 ± 6.45, 3.9–25.6 n = 5/5	6 ± 0.6, 5.4–6.6 n = 1/1
Histopathological grade	%, n = number of tumors	
- High;	61%, n = 14	64%, n = 7
- Low;	39%, n = 9	36%, n = 4
- No data available.	-	1
Maximum MIB-1 Proliferation Index	n = number of tumors	
<5/5/10/20/30/40/50/60/70/80 in %	1/5/1/5/1/2/2/3/1/1	1/1/1/2/1/1/1/0/0
Resection margins	%, n = number of tumors ***	
- R0;	70%, n = 16	58%, n = 7
- R1;	13%, n = 3	17%, n = 2
- R2.	17%, n = 4	25%, n = 3
Radiation (neo-/adjuvant/both) (no. of tumors)	0/6/1	0/2/0
Chemotherapy (neo-/adjuvant/both) (no. of tumors)	1/6/1	1/4/0

Note. No—number; SD—standard deviation; NF1—Neurofibromatosis Type 1; SPO—sporadic; SUV—standardized uptake value; PET—positron emission tomography using 18F-fluorodeoxyglucose (FDG). * Suspicion of malignancy—rapidly developing and severe symptoms/known and rapid tumor growth progression/large tumor size/MR radiological signs (inhomogeneity). ** SUVs > 4 in FDG PET were considered highly suspicious for malignancy [10]. *** Resection margins according to the residual tumor (R) classification [22].

**Table 2:** The difference in rating parameters between high-grade vs. low-grade and NF1 vs. sporadic MPNSTs.

**Variable**	***U***	**z**	***p***	**Mean Rank/Median** **High-Grade**	**Mean Rank/Median** **Low-Grade**
Preoperative MRC	154	−0.075	0.961	18.11/5, n = 19	17.88/5, n = 16
Postoperative MRC	169	−0.604	0.567	18.92/4, n = 19	16.91/4, n = 16
Preoperative SRS	140	−0.417	0.707	17.37/4, n = 19	18.75/4, n = 16
Postoperative SRS	153	−0.017	0.987	17.97/3, n = 19	18.03/3, n = 16
Preoperative VRS	155	−0.104	0.935	18.16/2, n = 19	17.81/2, n = 16
Postoperative VRS	171	−1.098	0.365	19/0, n = 18	15.81/0, n = 16
Max Proliferation Rate	166	−2.343	0.020	18.76/40, n = 17	11.23/20, n = 13
Preoperative Volume	149	−0.812	0.433	17.76/62.3, n = 17	15.07/112.2, n = 15
**Variable**	***U***	**z**	***p***	**Mean Rank/Median NF1**	**Mean Rank/Median Sporadic**
Preoperative MRC	107	−1.220	0.294	16.65/5, n = 23	20.58/5, n = 12
Postoperative MRC	126	−0.344	0.745	18.41/5, n = 23	17.21/3, n = 12
Preoperative SRS	109	−1.040	0.327	16.76/4, n = 23	20.38/4.5, n = 12
Postoperative SRS	146	−0.286	0.797	18.35/3, n = 23	17.33/3.5, n = 12
Preoperative VRS	223	−3.082	0.002	21.70/2, n = 23	10.92/0.5, n = 12
Postoperative VRS	155	−0.977	0.423	18.55/0, n = 22	15.58/0, n = 12
Max. Proliferation Rate	92	−0.213	0.836	15.7/20, n = 22	14.94/27.5, n = 8
Preoperative Volume	195	−3.456	<0.001	20.36/112.2, n = 22	8/11.6, n = 10

Note. *p* < 0.05. Significant *p* values between the two groups are written in bold. MRC—Medical Research Council Scale for Muscle Strength [18]; SRS—Sensory Rating Scale [19]; VRS—Verbal Rating Scale for measuring pain intensity [20]. The higher the mean rank values for motor and sensory classification and the lower the mean rank values for pain classification, the better the neurological status and the lower the pain intensity.

## Data Availability

The datasets analyzed during the current study are available from the corresponding author upon reasonable request.

## Supplementary Materials

The following supporting information can be downloaded at: https://www.mdpi.com/article/10.3390/cancers16223757/s1, Table S1: Immediate pre- and postoperative functional rating scores in (table above) 23 NF1-associated and (table below) 12 sporadic malignant peripheral nerve sheath tumors; Table S2: Detailed patient/tumor characteristics; Table S3: Detailed clinical status over the long-term treatment course; Figure S1: Scatterplot to assess linearity between the preoperative tumor volume and the maximum value of the MIB-1 proliferation index.
