# Supplementary material for: Perioperative Observations and Outcome in Surgical Treatment of Malignant Peripheral Nerve Sheath Tumors
Source: Cancers (Basel). 2024 Nov 7;16(22):3757. doi: 10.3390/cancers16223757 (PMC11592335; doi:10.3390/cancers16223757)
Supplement: Supplementary file 1 [file cancers-16-03757-s001.zip › cancers-3247354-supplementary.pdf]

Supplementary Table S1. Immediate pre-and postoperative functional rating scores in (table above) 23 NF1-associated and (table below) 12 sporadic operated malignant peripheral nerve sheath tumors.

**MRC Scale**			**Postoperative Class (No)**			
**Preoperative Class (No)**	**0**	**1**	**2**	**3**	4	5
0 (1)	1	0	0	0	0	0
1 (3)	0	0	2	0	1	0
2 (2)	0	0	0	1	1	0
3 (0)	0	0	0	0	0	0
4 (5)	1	0	0	0	3	1
5 (12)	3	0	0	0	2	7
**SRS Scale**			**Postoperative Class (No)**			
Preoperative Class (No)	0	1	2	3	4	5
0 (0)	0	0	0	0	0	0
1 (7)	0	5	0	1	0	1
2 (2)	0	0	0	2	0	0
3 (2)	1	0	0	0	0	1
4 (3)	2	0	0	0	1	0
5 (9)	0	1	0	1	1	6
**VRS Scale**			**Postoperative Class (No)**			
Preoperative Class (No)	0	1	2	3	-	-
0 (3)	3	0	0	0		
1 (2)	1	1	0	0		
2 (8)	4	4	0	0		
3 (10)	5	3	1	1		

**MRC Scale**			**Postoperative Class (No)**			
**Preoperative Class (No)**	**0**	**1**	**2**	**3**	4	5
0 (0)	0	0	0	0	0	0
1 (0)	0	0	0	0	0	0
2 (2)	1	0	1	0	0	0
3 (1)	0	0	1	0	0	0
4 (0)	0	0	0	0	0	0
5 (9)	2	0	0	0	1	6
**SRS Scale**			**Postoperative Class (No)**			
Preoperative Class (No)	0	1	2	3	4	5
0 (1)	1	0	0	0	0	0
1 (0)	0	0	0	0	0	0
2 (1)	0	0	1	0	0	0
3 (1)	0	0	0	1	0	0
4 (3)	2	0	0	0	1	0
5 (6)	0	0	0	0	1	5
**VRS Scale**			**Postoperative Class (No)**			
Preoperative Class (No)	0	1	2	3	-	-
0 (6)	6	0	0	0		
1 (2)	1	1	0	0		
2 (4)	2	1	1	0		
3 (0)	0	0	0	0		

MRC Scale: Medical Research Council Scale for Muscle Strength (MCR)[1]; **SRS Scale:** sensory rating scale (SRS)[2]; **VRS Scale:** four-point Verbal Rating Scale (VRS)[3] with the words and Scoring in brackets “no pain”

(0), “slight pain” (1), “moderate pain” (2), and “severe pain” (3) for measuring pain intensity. The immediate pre- and postoperative status was evaluated directly before and after surgery during the in-patient stay.

Supplementary Table S2. Detailed patients/tumor characteristics.

Patient No	Tumor No	Sex	Disease Type	Family History for NF1	*NF1* Mutation Type	Preop biopsy	Age at time of surgery in years	Degree of resection (R0-2)*	Maximum Proliferation index (in %)	Histological Grading high vs. low	Location of metastasis	Radiation	Chemo-therapy	PET MRI/CT SUV	Direct preop tumor volume in cm^3	Overall survival in months
1	1	M	NF1	no	MIS	no	54	R0	5,00	low	-	-	-	-	29,4	
2	2	M	NF1	no	NS	no	48	R2	20,00	high	-	adjuvant	-	-	108,9	14,03
3	3	M	NF1	no	SP	yes	19	R0	60,00	high	cerebral	-	adjuvant, Trametinib	14	2294,3	9,43
4	4	F	NF1	yes	-	no	10	R0	5,00	low	-	-	-	3,9	66,6	
5	5	F	NF1	no	-	yes	12	R0	60,00	high	-	-	neoadjuvant, Doxorubicin/ Ifosfamide	7,6	844	11,93
6	6	M	NF1	no	-	no	16	R0	-	high	-	-	-	-	115,5	3,74
7	7	F	NF1	no	-	no	21	R1	20,00	low	-	adjuvant	adjuvant, Doxorubicin/ Ifosfamide	-	24,5	
8	8	M	NF1	no	-	yes	51	R2	40,00	high	-	adjuvant	adjuvant, Doxorubicin/ Ifosfamide	-	281,1	7,75
9	9	F	NF1	yes	NO	no	21	R0	70,00	high	upper leg	-	-	18,3	371,8	10,75
	10					no	22	R0	80,00	high	cerebral	adjuvant	-	7,2	-	
10	11	M	NF1	yes	MIS	yes	25	R0	5,00	low	-	-	-	5,8	170,6	
11	12	M	NF1	no	NO	no	8	R0	10,00	high	-	-	-	-	32,3	
12	13	M	NF1	yes	SP	no	47	R1	5,00	low	-	-	-	-	13,5	
13	14	M	NF1	yes	DEL	no	30	R0	20,00	low	-	-	-	7,5	254,7	
	15					no	36	R0	3,00	high	-	-	-	3,9	12,039	
14	16	M	NF1	yes	-	no	38	R1	5,00	high	sacral	adjuvant	adjuvant, Doxorubicin/ Ifosfamide	-	62,3	7,43

15	17	F	NF1	yes	NO	no	33	R0	50,00	high	-	-	adjuvant, Doxorubicin/ Ifosfamide	-	99,4	
16	18	F	NF1	no	NS	yes	27	R2	60,00	high	meningeosis, thoracic	adjuvant	neoadjuvant + adjuvant, Doxorubicin/ Ifosfamide	25,6	201,3	11,73
17	19	M	NF1	no	-	yes	34	R0	20,00	low	pulmonary	-	-	-	908,3	
18	20	M	NF1	no	-	no	26	R0	50,00	high	lumbar spine	neoadjuvant + adjuvant	adjuvant, Doxorubicin/ Olaratumab	-	177,4	5,98
19	21	F	NF1	no	-	no	31	R2	40,00	high	-	-	-	-	14,3	20,54
20	22	M	NF1	no	-	yes	18	R0	20,00	high	-	-	-	-	11,5	
21	23	M	NF1	no	-	no	28	R0	30,00	low	-	-	-	6,1	486,6	
22	24	M	SPO	no	-	no	56	R2	50,00	high	hepatic, pulmonary, ossary	adjuvant	adjuvant, Doxorubicin/ Olaratumab	-	26,4	13,24
23	25	F	SPO	no	-	no	28	R0	5,00	low	-	-	-	-	2,5	
24	26	M	SPO	no	-	no	67	R0	-	high	lymph node inguinal	adjuvant	adjuvant, Doxorubicin/ Ifosfamide	6,6	-	15,84
25	27	M	SPO	no	-	no	45	R2	60,00	high	-	adjuvant	-	-	44,5	6,67
26	28	M	SPO	no	-	yes	14	R0	-	low	-	-	adjuvant, Doxorubicin/ Ifosfamide	-	3,2	
27	29	F	SPO	no	NO	yes	39	R2	25,00	high	-	adjuvant	-	-	12,5	
28	30	F	SPO	no	-	no	35	R1	40,00	high	pulmonary	adjuvant	adjuvant, Doxorubicin/ Ifosfamide	-	21,5	
29	31	F	SPO	no	-	no	34	R0	-	-	-	-	-	-	10,7	
30	32	M	SPO	no	-	yes	62	R0	30,00	high	-	neoadjuvant	neoadjuvant, Doxorubicin/ Ifosfamide	5,4	44,9	
31	33	M	SPO	no	-	yes	38	R0	3,00	low	-	-	-	-	-	
32	34	F	SPO	no	-	yes	55	R1	10,00	high	-	adjuvant	-	-	10,483	37,01

33	35	M	SPO	no	-	yes	31	R0	-	low	-	-	-	-	1,2	

No – Number; **M** – Male; **F** – Female; **MIS** – missense mutation; **DEL** – deletion; **SP**- splicing mutations; **NS** – nonsense mutation; **NO** – no mutation detected; **NF1** – Neurofibromatosis type 1; **SPO** – sporadic; **Preop** – preoperative; Preop biopsy – either in form of a needle or open biopsy; *Resection margins according to the residual tumor (R) classification; R0/R1 Classification System (R0=no residual tumor, R1=microscopic residual tumor, R2 =macroscopic residual tumor)[4]; **PET** — positron emission tomography using FDG (grey-shaded are the MRI FDG PET); **MRI** – magnet resonance imaging; **CT** – computertomography.

Supplementary Table S3. Detailed clinical status in the long-term course.

		MRC Scale		Motor Function Status	SRS Scale		Sensory Function Status	VRS Scale		Pain Rating Status	Last follow-up visit at our NF Center	
**Patient No**	**Tumor No**	**Direct preop**	**Direct postop**	**Direct postop**	**Direct preop**	**Direct postop**	**Direct postop**	**Direct preop**	**Direct postop**	**Direct postop**	**Postop in month**	**Clinical Status***
1	1	5	5	0	5	5	0	2	0	1	85	2
2	2	2	3	1	1	1	0	2	1	1	0**	-
3	3	4	4	0	5	5	0	2	1	1	7	2
4	4	4	4	0	1	1	0	3	0	1	67	0
5	5	5	0	2	1	1	0	3	0	1	1	2
6	6	5	4	2	5	3	2	2	1	1	1	2
7	7	4	5	1	1	3	1	3	2	1	71	0
8	8	4	4	0	1	1	0	3	0	1	0**	-
9	9	1	4	1	5	5	0	0	0	0	12	1
	10	1	4	1	1	5	1	0	0	0	7	1
10	11	5	0	2	5	1	2	3	0	1	87	1
11	12	5	5	0	5	5	0	2	1	1	78	2
12	13	5	5	0	3	5	1	3	0	1	63	2
13	14	1	2	1	2	3	1	3	2	1	72	0
	15	1	2	0	2	3	0	3	3	0	6	1
14	16	5	4	2	5	4	2	2	0	1	3	2
15	17	5	5	5	5	5	5	2	0	1	31	2
16	18	0	0	0	1	1	0	3	-	-	6	2
17	19	5	0	2	3	0	2	1	0	1	165	0
18	20	4	0	2	4	0	2	1	1	0	3	2
19	21	5	5	0	4	4	0	3	2	1	7	0
20	22	5	5	0	4	0	2	2	0	1	10	0
21	23	5	5	0	5	5	0	0	0	0	12	0
22	24	5	5	0	5	5	5	0	0	0	5	2

23	25	5	4	4	5	4	2	1	0	1	85	1
24	26	5	5	0	5	5	0	0	0	0	2	0
25	27	5	0	2	4	0	2	1	1	0	0**	-
26	28	5	5	0	5	5	0	0	0	0	115	0
27	29	5	0	2	4	0	2	2	2	0	9	0
28	30	3	2	2	4	4	0	0	0	0	107	0
29	31	5	0	2	5	0	2	2	0	1	0**	-
30	32	2	2	0	3	3	0	2	0	1	43	2
31	33	2	0	2	2	2	0	2	1	1	69	0
32	34	5	5	0	0	0	0	0	0	0	11	0
33	35	5	5	0	5	5	0	0	0	0	77	0

Preop – preoperative; **Postop** – postoperative; **Status:** equal=0, improved=1, worsened=2; * Each change of values in one category was documented as part of the last clinical examination and based on the immediate (direct) postoperative status during the in-patient stay. ** In these four patients merely the resection was performed at our Center of Neurofibromatosis and further treatment was planned/performed at local institutions.

Supplementary Figure S4. Scatterplot to assess linearity between the preoperative tumor volume and the maximum value of MIB-1 proliferation index.

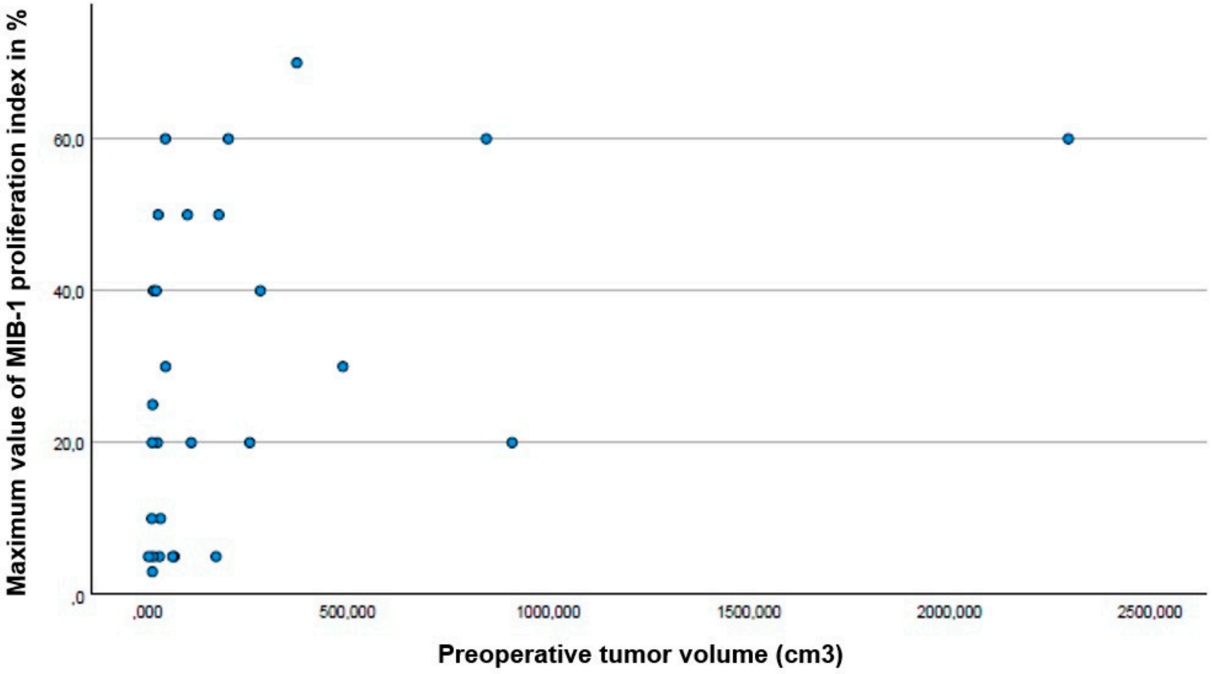

References

1. Compston, A. Aids to the investigation of peripheral nerve injuries. Medical Research Council: Nerve Injuries Research Committee. His Majesty's Stationery Office: 1942; pp. 48 (iii) and 74 figures and 7 diagrams; with aids to the examination of the peripheral nervous system. By Michael O'Brien for the Guarantors of Brain. Saunders Elsevier: 2010; pp. [8] 64 and 94 Figures. *Brain* **2010**, *133*, 2838-2844, doi:10.1093/brain/awq270.
2. Zipfel, J.; Al-Hariri, M.; Gugel, I.; Haas-Lude, K.; Grimm, A.; Warmann, S.; Krimmel, M.; Mautner, V.F.; Tatagiba, M.; Schuhmann, M.U. Surgical management of peripheral nerve sheath tumours in children, with special consideration of neurofibromatoses. *Childs Nerv Syst* **2020**, *36*, 2433-2442, doi:10.1007/s00381-020-04703-6.

3. Lee, H.J.; Cho, Y.; Joo, H.; Jeon, J.Y.; Jang, Y.E.; Kim, J.T. Comparative study of verbal rating scale and numerical rating scale to assess postoperative pain intensity in the post anesthesia care unit: A prospective observational cohort study. *Medicine (Baltimore)* **2021**, *100*, e24314, doi:10.1097/MD.0000000000024314.
4. Hermanek, P.; Wittekind, C. The pathologist and the residual tumor (R) classification. *Pathol Res Pract* **1994**, *190*, 115-123, doi:10.1016/S0344-0338(11)80700-4.
